# Toll-like receptor 3 (TLR3) promotes the resolution of *Chlamydia muridarum* genital tract infection in congenic C57BL/6N mice

**DOI:** 10.1371/journal.pone.0195165

**Published:** 2018-04-06

**Authors:** Sebastian E. Carrasco, Sishun Hu, Denise M. Imai, Ramesh Kumar, George E. Sandusky, X. Frank Yang, Wilbert A. Derbigny

**Affiliations:** 1 School of Veterinary Medicine and Comparative Pathology Laboratory, University of California-Davis, Davis, California, United States of America; 2 Department of Microbiology and Immunology, Indiana University School of Medicine, Indianapolis, Indiana, United States of America; 3 College of Veterinary Medicine, Huazhong Agricultural University, Wuhan, People’s Republic of China; 4 Department of Pathology, Indiana University School of Medicine, Indianapolis, Indiana, United States of America; University of the Pacific, UNITED STATES

## Abstract

*Chlamydia trachomatis* urogenital serovars primarily replicate in epithelial cells lining the reproductive tract. Epithelial cells recognize *Chlamydia* through cell surface and cytosolic receptors, and/or endosomal innate receptors such as Toll-like receptors (TLRs). Activation of these receptors triggers both innate and adaptive immune mechanisms that are required for chlamydial clearance, but are also responsible for the immunopathology in the reproductive tract. We previously demonstrated that *Chlamydia muridarum* (*Cm*) induces IFN-β in oviduct epithelial cells (OE) in a TLR3-dependent manner, and that the synthesis of several cytokines and chemokines are diminished in *Cm*-challenged OE derived from TLR3^-/-^ 129S1 mice. Furthermore, our *in vitro* studies showed that *Cm* replication in TLR3^-/-^ OE is more efficient than in wild-type OE. Because TLR3 modulates the release inflammatory mediators involved in host defense during *Cm* infection, we hypothesized that TLR3 plays a protective role against *Cm*-induced genital tract pathology in congenic C57BL/6N mice. Using the *Cm* mouse model for human *Chlamydia* genital tract infections, we demonstrated that TLR3^-/-^ mice had increased *Cm* shedding during early and mid-stage genital infection. In early stage infection, TLR3^-/-^ mice showed a diminished synthesis of IFN-β, IL-1β, and IL-6, but enhanced production of IL-10, TNF-α, and IFN-γ. In mid-stage infection, TLR3^-/-^ mice exhibited significantly enhanced lymphocytic endometritis and salpingitis than wild-type mice. These lymphocytes were predominantly scattered along the endometrial stroma and the associated smooth muscle, and the lamina propria supporting the oviducts. Surprisingly, our data show that CD4^+^ T-cells are significantly enhanced in the genital tract TLR3^-/-^ mice during mid-stage *Chlamydial* infection. In late-stage infections, both mouse strains developed hydrosalpinx; however, the extent of hydrosalpinx was more severe in TLR3^-/-^ mice. Together, these data suggest that TLR3 promotes the clearance of *Cm* during early and mid-stages of genital tract infection, and that loss of TLR3 is detrimental in the development hydrosalpinx.

## Introduction

*Chlamydia trachomatis* (*Ct*), a Gram-negative obligate intracellular bacterium, is the leading cause of bacterial sexually transmitted diseases worldwide with an estimated incidence of 105.7 million infections in adults every year [1]. Although *Ct* infections in women are treatable with antibiotics, these infections can remain largely asymptomatic and go undetected in approximately 70–80% of the cases [2]. *Chlamydia trachomatis* infections in the reproductive tract of women can also lead to cervicitis and endometritis as well as to the development of serious complications, including pelvic inflammatory disease (PID), tubal scarring and infertility, fallopian tube blockage with serous fluid (hydrosalpinx), chronic pelvic pain, and ectopic pregnancy [2, 3]. Clearance of *Chlamydia* needs the coordinated action of both the innate immune response and host CD4^+^ T-cells, which together are essential for the optimal resolution of primary chlamydial genital infections in mice. [3–5]. However, *Chlamydia* infection causes the induction of a specific subset of innate inflammatory mediators and the recruitment of CD8^+^ T-cells into the female genital tract, and these factors are known to have a significant role in development of genital tract pathology [6–8]. The ultimate goal of the continued research on *Ct* pathogenesis is to identify immune mediators that generate long-term protective immune responses against *Ct* infections, and to ascertain immune targets that modulate the immune responses leading to upper reproductive tract pathology.

The murine model of *Chlamydia muridarum* genital tract infection recapitulates many aspects of the pathogenesis and immunity associated with *Ct* genital tract infections in women [9]. The initial immune response in the genital tracts of mice infected with *C*. *muridarum* is dominated by myeloid cell infiltrates, including neutrophils, which are predominantly recruited to the cervical epithelium [8, 10]. As infection progresses in mice, *Chlamydia* disseminates to epithelial surfaces lining the uterine horns and oviducts, which become infiltrated by CD4^+^ and CD8^+^ T-cells, plasma cells, and macrophages [9, 10]. Late stages of *C*. *muridarum* infection in mice can then lead to hydrosalpinx, fibrosis and/or infertility [4, 11, 12], which are also common post-infection sequelae in women. There are multiple inflammatory cells infiltrating the genital tract of mice throughout the course of *Cm* infection; however, none of them are the primary target of infection. Instead, epithelial cells are the primary targets as *Chlamydia* selectively replicates within the reproductive tract epithelium, and these cells are also critical on initiating and propagating the immune response during infection [13, 14].

As an obligate intracellular pathogen, *Chlamydia* species are known to interact with host-cell pattern recognition receptors (PRRs), including Toll-like receptors (TLRs) and a variety of other intracellular cytosolic receptors [13]. Activation of these receptors triggers the secretion of pro-inflammatory cytokines and chemokines, which in succession induces the recruitment of inflammatory cells. Chlamydial LPS and heat shock proteins (HSPs) are ligands for TLR4 [15–17], while chlamydial plasmid-regulated ligands and peptidoglycans are ligands for TLR2 in phagocytes [13, 18, 19]. Several other types of intracellular sensors have been shown to play role in the recognition of *Chlamydia* in phagocytes, including the intracellular nucleotide sensors cyclic GMP–AMP (cGAMP) synthase (cGAS), stimulator of interferon genes (STING), nucleotide-binding oligomerization domain-containing 1 (NOD1), and NLR family pyrin domain containing 3 (NLRP3) inflammasome. [13, 20, 21].

TLR3 is a receptor for double-stranded RNA (dsRNA) and is known to activate transcription of IFN-β via the adaptor protein Toll-IL-1 receptor (TIR) domain-containing adaptor molecule-1 (TICAM-1) [also called TIR-domain-containing adapter inducing IFN-β (TRIF)] [22, 23]. TLR3 has been identified as the major MyD88-independent PRR stimulated in the type-1 IFN responses to many different viral infections due to its intracellular localization [24]; however, its role in bacterial infection is poorly understood. Although a double-stranded RNA moiety for *Chlamydia* has not yet been identified as part of chlamydial structure, we previously showed that *C*. *muridarum* infected murine oviduct epithelial (OE) cells secrete IFN-β in a largely TLR3 dependent manner, and that OE cells deficient in TLR3 showed dramatic reductions in the synthesis of other inflammatory immune mediators in addition to IFN-β [25, 26]. Our previous data also showed IL-6 and CCL5 synthesis was diminished during early stages of *Cm* infection in the genital tract of TLR3^-/-^ mice from a 129S1 genetic background [26]. Furthermore, *C*. *muridarum* replication in murine OE cells is more robust in the absence of TLR3, suggesting that activation of TLR3 negatively affects *Chlamydia* growth. Because TLR3 modulates the release of inflammatory mediators involved in host defense *in vitro* as well as during *Cm* genital tract infection in mice, we hypothesized that TLR3 plays a protective role against *Cm*-induced genital tract pathology in congenic C57BL/6N mice.

In this study, we further examine the role of TLR3 in the development of genital tract pathology during chlamydial infection. Using the *Cm* model of chlamydial genital tract infection, we infected wild-type and TLR3^*-/-*^ mice, and compared the vaginal synthesis of cytokines and chemokines, *Chlamydia* burden, CD4^+^/CD8^+^ T-cell profiles, and genital tract pathology at different time points throughout infection. We found that TLR3 was critical in the synthesis of several intravaginal pro-inflammatory cytokines such as IFN-β, IL-1β, and IL-6, and had an altered synthesis of IL-10, TNF-α, and IFN-γ during early stages of *Cm* genital tract infection. TLR3 also played a role in host defense as TLR3^-/-^ mice had significantly higher levels of genital tract chlamydial shedding than wild-type mice during the first three weeks of genital tract infection. Furthermore, TLR3^-/-^ mice exhibited significantly higher lymphocytic endometritis and salpingitis than wild-type mice during mid-stages of genital tract infection. Interestingly, the presence of CD4^+^ T-cells were markedly enhanced in the genital tract of TLR3^-/-^ mice at this stage of *Chlamydia* infection. Finally, TLR3^-/-^ mice exhibited a significantly increased frequency in the development of hydrosalpinx than wild-type mice during late-stages of genital tract infection, suggesting that TLR3 had a role in preventing the development of hydrosalpinx *in vivo*.

## Materials and methods

### Ethics statement

C57BL/6J (control) and TLR3-deficient mice were purchased from The Jackson Laboratory (Bar Harbor, ME) at 6–8 weeks of age. All mice were provided food and water *ad libitum*, and kept on a standard 12-hour light/dark cycle. All mice were given at least a 1-week period where they were allowed to acclimate to their new environment. After acclimation, female C57BL/6NJ and TLR3-deficient mice were injected with 2.5 mg Depo-Provera after being briefly anesthetized with isoflurane prior to any experiment, and all mice were allowed to recover for no less than 1-week after the Depo-Provera treatment. To alleviate any possible distress, the mice were also briefly anesthetized with isoflurane prior to either intravaginal infection with *C*. *muridarum*, the insertion of calcium alginate swabs, or the insertion of aseptic vaginal sponges. All mice were monitored daily for lethargy, signs of vaginal bleeding, and/ or death. None of the mice exhibited morbidity during the course of these studies. All mice were euthanized by either exposure to isoflurane or inhalation of carbon dioxide, followed by exsanguination. The Indiana University Institutional Animal Care and Use Committee (IACUC) approved all experimental animal protocols. All care was used to ensure that steps were taken to ameliorate animal suffering in all work involved in the removal of genital-tract tissue.

### Chlamydia stocks

*Mycoplasma*-free *C*. *muridarum* Nigg, previously known as *C*. *trachomatis* strain (MoPn), was grown and titrated in McCoy cells (ATCC) as described [27, 28]. Elementary bodies were harvested from infected cells, resuspended in SPG buffer (250mM sucrose, 10mM sodium phosphate, and 5mM L-glutamic acid, pH 7.2), and quantified on McCoy cells using methodology described previously [26, 27, 29].

### Mice and infections

Wild-type control mice C57BL/6NJ [Stock No 005304] and TLR3-deficient mice B6N.129S1-Tlr3tm1^Flv^/J [Stock No 009675] were purchased from The Jackson Laboratory at 6–8 weeks of age. This TLR3 deficient mouse strain is a fully congenic on the C57BL/6N genetic background. Briefly, this particular strain of TLR3 deficient mice were the result of a backcross into C57BL/6J F1 hybrids that were self-crossed to generate F2 hybrids identified as homozygous with or without the TLR3 deletion. The homozygous mice are maintained at The Jackson Laboratory by breeding descendants of the original homozygous F2 hybrids. All mice were housed in Indiana University Purdue University-Indianapolis specific pathogen-free (SPF) facilities. Age-matched mice were used for the experiments in this study. The Indiana University Institutional Animal Care and Utilization Committee (IACUC) approved all experimental protocols.

Infections of mice were done as described in [18] with some minor modifications. Mice were treated with 2.5 mg of Depo-Provera (medroxyprogesterone acetate; Pfizer, New York, NY) in 0.1 ml saline to synchronize the estrous cycle and increase their susceptibility to a chlamydial infection, one week before vaginal infection with 10^5^ IFU *C*. *muridarum* (approximately 100 times the ID50) in 10μl sucrose-phosphate-glutamic acid (SPG) buffer. The infection was monitored by swabbing the vaginal vault and cervix with a calcium alginate swab (Spectrum Medical Industries, Los Angeles, CA) and determining the number of inclusion forming units (IFU) recovered from the swabs by plating serial dilutions on McCoy cell monolayers as described [27, 28, 30]. Briefly, swabs were suspended in DMEM/ 10% FCS supplemented with vancomycin, gentamicin, and cycloheximide and then vortexed for 1 min in the presence of glass beads to release chlamydial organisms from the swab. Individual wells of McCoy cell monolayers in 96-well plates were inoculated with serial dilutions of each sample in a total volume of 200μl/well, followed by centrifugation at 1200 rpm (200 × g) in a table-top centrifuge for 1 hour. After centrifugation, the plates were then incubated at 37°C in a 5% CO2 humidified incubator for 2h before removing supernatants and replacing it with fresh medium. Plates were returned to the incubator for an additional 32h. Afterwards, the plates were removed from the incubator, supernatants removed, and the monolayers were washed with PBS before being fixed with methanol. Chlamydial inclusions were identified by the addition of anti-*Chlamydia* immune sera and anti-mouse immunoglobulin G (IgG) conjugated to fluorescein-isothiocyanate secondary antibody (ICN Immunobiologicals, Irvine, Calif.). The number of inclusion bodies within 20 fields was counted under a fluorescent microscope, and IFU/ml were calculated. Each mouse strain (wildtype and TLR3-deficient) were infected in groups of five, and each experiment was repeated at least three times.

### Determination of cytokine production

Vaginal swab samples for measurement of genital secretions of innate-immune cytokines and chemokines were collected via the aseptic sponge technique [18]. Aseptic surgical sponges (ear wicks, 2 x 5 mm) (DeRoyal, Powell, TN) were inserted into the vaginas of anesthetized *Chlamydia*-infected and mock infected mice, each day during the first 21 days post-infection. Each sponge was retrieved 30 min later and individually collected in microfuge tubes for storage at -80°C. The sponges were then soaked in 300μl of sterile resuspension buffer (PBS supplemented with 0.5% BSA and 0.05% TWEEN 20) for one hour on ice, and filtered through a pre-blocked 0.2-μm cellulose acetate filter by centrifugation in a Spin-X microfuge tube (Fisher Scientific, Pittsburgh, PA) prior to analysis. The filtrates were frozen at -80°C and shipped on dry-ice to Aushon Biotechnology (Billerica, MA) for multiplex analyses. The analyses of cytokine and chemokine synthesis was performed by Aushon using their in-house Ciraplex^™^ multiplex assay. The samples were analyzed in triplicate, and the assays were conducted on all three replicates of mouse infections.

### Tissue processing for isolation of lower genital tract and splenic T-cells

Genital tracts were excised from each group of wild-type and TLR3-deficient mice sacrificed at day 7 and day 21 post-infection. Each mouse was handled separately, and the lower portion of the genital tract of each mouse (defined as the area from cervix to the start of uterine the horn) was separated from the rest of the genital tract as previously described [31]. The lower genital tracts were minced in RPMI-1640 medium supplemented with 10% FBS (Sigma-Aldrich, St. Louis, MO) and filtered throw a 70μm nylon mesh cell strainer. The resulting slurry was then digested with freshly prepared collagenase I solution in RPMI-1640 supplemented with 10% FBS (1mg/ml; Sigma-Aldrich, St. Louis, MO) for 1h at 37 °C to obtain a single-cell suspension. The lymphocytes were isolated using Percoll^™^ gradient (density 1.129g/ml, GE Healthcare Bio-sciences AB, Sweden) as described in the protocol [32]. Spleens from individual mouse were minced and filtered through a 70μm nylon mesh cell strainer. Red blood cells (RBC) were lysed with 1x RBC lysis solution (eBioscience, SanDiego CA) for 5 min at room temperature. Total splenocytes were re-suspended in RPMI-1640 medium supplemented with 10% FBS after centrifugation.

### Flow cytometry

Isolated murine genital tract and splenic T-cells were initially blocked for 20 min in 10% rabbit serum (Sigma-Aldrich, St. Louis, MO). Following blocking for non-specific binding, 1×10^6^ cells were stained with anti-mouse CD3-PE-Cy7 (clone 17A2, BioLegend, San Diego CA), CD4-FITC (Ref# MCD0401, Invitrogen, Carlsbad CA) and CD8a-PE (Invitrogen) for 30 min at 4°C. Isotype control antibodies FITC Rat IgG2λ κ (clone RTK2758), PE Rat IgG2b κ (clone RTK4530), and PE-Cy7 Rat IgG2b κ (clone RTK4530) used in these experiments were obtained from BioLegend. Cell population analysis was conducted by LSR4 flow cytometer equipped with BD FACSDiva software (BD Biosciences, Franklin Lakes, NJ). A lymphocyte specific gating was set according to forward and side scatters profiles.

### Histopathology

Genital tracts were extracted *en bloc* from mice sacrificed on day 7, 21, or 56 post-infection and then fixed in 4% paraformaldehyde for 48 hrs. Paraffin-embedded, 4-μm sections containing cervix and both uterine horns and oviducts were stained with hematoxylin and eosin (H&E). Genital tract sections (cervix, uterine body and horns, and oviducts) were independently examined and graded for the presence of acute inflammation (neutrophilic infiltrates), chronic inflammation (lymphocytic infiltrates), epithelial erosion and disruption, and luminal distension of uterine horns and oviducts using a previously described four-tiered semiquantitative scoring system [33, 34]. Inflammatory cell infiltrates or epithelial erosion and disruption were scored as follows: 0, normal; 1, occasional foci or minimal increase in the parameter; 2, scattered (2 to 4) aggregates or mild focal to multifocal increase in parameter; 3, numerous aggregates (< 4) or moderate multifocal to coalescing increase in parameter; 4, numerous, dense or confluent aggregates or severe diffuse or multifocal to coalescing increase in parameter. The uterine horn dilation and oviduct hydrosalpinges were assessed grossly and microscopically, and scoring of these parameters were performed using a semiquantitative scoring system described previously [34]. The dilation of the uterine horns and oviducts (hydrosalpinges) were scored as follows: 0, normal; 1, mild increase in dilatation of a single segment of these parameters; 2, one to three, unilateral or bilateral, dilated segments of these parameters; 3, more than three, unilateral or bilateral, dilated segments of these parameters; and 4, confluent pronounced or marked unilateral or bilateral dilatation of uterine horns and oviducts. All scoring was performed in a double-blinded fashion with regards to the treatment groups, and scores for each evaluated parameter were combined and averaged to provide one score per animal.

### Immunohistochemistry (IHC)

Paraffin-embedded sections were processed at the UC Davis Center for Genomic Pathology Laboratory for immunohistochemical staining of CD4 and CD8 T-cells in the genital tracts of a subset uninfected and infected mice as previously described [35]. Sections of spleen were used as internal control. Rat monoclonal anti-mouse CD4 (clone RM4-5, BD Biosciences) and rat monoclonal anti-mouse CD8 (clone 53–6.7, Biolegend) were individually diluted in 0.5% bovine serum albumin (BSA) in PBS (pH 7.4) and incubated with tissue sections for 1 hr at room temperature. The slides were then rinsed twice with PBS buffer for 5 min each. Immunohistochemical reaction was performed by using a VECTASTAIN Elite ABC kit (Vector Laboratories, Burlingame, CA) with biotinylated anti-rat IgG (Vector Laboratories) following manufacturer’s protocol. After the slides were washed twice with PBS, sections were then incubated in 0.1% 3,3’-diaminobenzidine (DAB) solution (Dako, Carpinteria, CA) following the manufacturer’s directions. Paraffin-embedded sections from uninfected and infected mice (n = 3 per group) were processed at the California Animal Health and Food Safety Laboratory System for immunohistochemical detection of *Chlamydia* LPS antigen in genital tracts of these mice s previously described [36]. Sections of liver from a *Chlamydia* infected bird was used as an internal control for positive reaction.

### Statistical analysis

Statistical comparison between the two experimental groups for the vaginal cytokines levels and the CD4—CD8 counts in tissues over the course of infection were made by a Student’s *t*-test with Welch correction. For analysis of histopathology scores, the Mann-Whitney rank sum test was performed to assess differences in inflammation between the two experimental groups on three separate time points of infection. A *p* value ≤ 0.05 was considered statistically significant. Analyses were performed using GraphPad Prism v7 statistical program (GraphPad Software, San Diego, CA).

## Results

### *Chlamydia muridarum* shedding is more robust in the genital tracts of TLR3-deficient C57BL/6 mice

**Fig 1:**
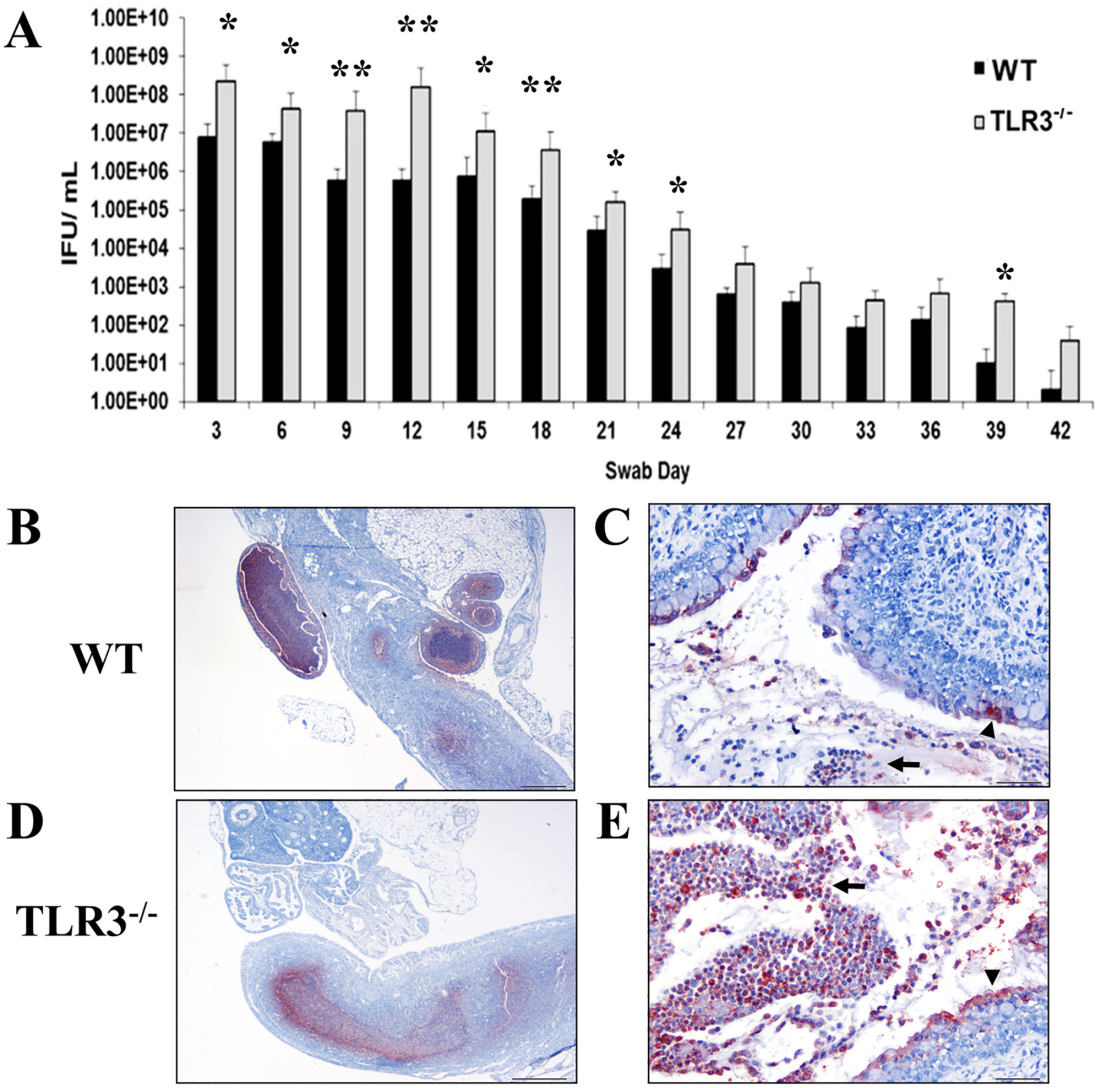
The levels of *Chlamydia muridarum* shedding from genital tracts of TLR3^-/-^ mice are significantly higher during the first 24 days of infection. **A)** Genital tract infections were performed, swabs were collected every third day for 42 days, and *C*. *muridarum* titers were determined by infecting fresh McCoy cell monolayers (see Materials and methods). Representative data from an average of 5 mice are shown. IFU = inclusion forming units. * = *p* <*0.05*; ** = *p* <*0.005*. **B-E)** Immunostaining of *Chlamydia muridarum* in genital tracts to wild-type (WT) and TLR3^-/-^ mice at day 7 post-challenge. Detection of *Chlamydia* antigen (red staining) within neutrophils (arrow) and epithelial cells (arrowhead) in oviducts (B), endometrium (C), and cervix from a representative WT mouse. Detection of *Chlamydia* antigen (red staining) associated with neutrophils (arrows) and epithelial cells (arrowhead) in endometrium and cervix (D and E), from a representative TLR3^-/-^ mouse.

We previously showed that TLR3^-/-^ mice bred in the 129S1 background exhibited significantly higher levels of chlamydial shedding from the genital tract when compared to wild-type mice within the first four weeks of infection [25, 26]. Additionally, our previous studies showed that *Chlamydia* replication in oviduct epithelial cells (OE) deficient in TLR3 is more efficient than the 129S1 wild-type OE cells. The data from these experiments also demonstrated that TLR3 had a biological impact on the innate immune response to *Chlamydia* infection in mice; however, the exact effect that TLR3 signaling had on the genital tract pathology of *C*. *muridarum* infection in mice was unclear. To determine the biological significance that TLR3 had on chlamydial shedding in mice bred in the C57BL/6N genetic background, we infected groups of five wild-type and five C57BL/6N TLR3^-/-^ (referred as TLR3^-/-^) intravaginally with 10^5^ IFU *C*. *muridarum*. As shown in Fig 1A, TLR3^-/-^ mice bred in the C57BL/6 genetic background mice shed *Chlamydia* at significantly higher levels throughout the first 24 days of infection than did wild-type mice. Similar to our previous data using 129S1 mice, the chlamydial titers for both C57BL/6N strains were virtually the same after day 24 and for the remainder of infection, and the clearance kinetics were similar between both strains. To illustrate the localization of *C*. *muridarum* in the genital tract of mice, we performed immunohistochemistry in a subset of genital tracts of mice at day 7 of infection (Fig 1B–1F). *Chlamydia* antigen was detected in the cytoplasm of epithelial cells lining the oviducts as well as the endometrium of the uterine horns and cervical regions. There was also *Chlamydia* antigen commonly associated with degenerate and necrotic neutrophils present in the lumina of the genital tracts of wild-type and TLR3^-/-^ mice (Fig 1B). Collectively, our data suggest that TLR3 controls *C*. *muridarum* replication during early and middle genital tract infection in both C57BL/6N and 129S1 mouse strains.

### TLR3 deficiency in mice results in increased chronic endometritis and salpingitis during the middle stages of *C*. *muridarum* infection

**Fig 2:**
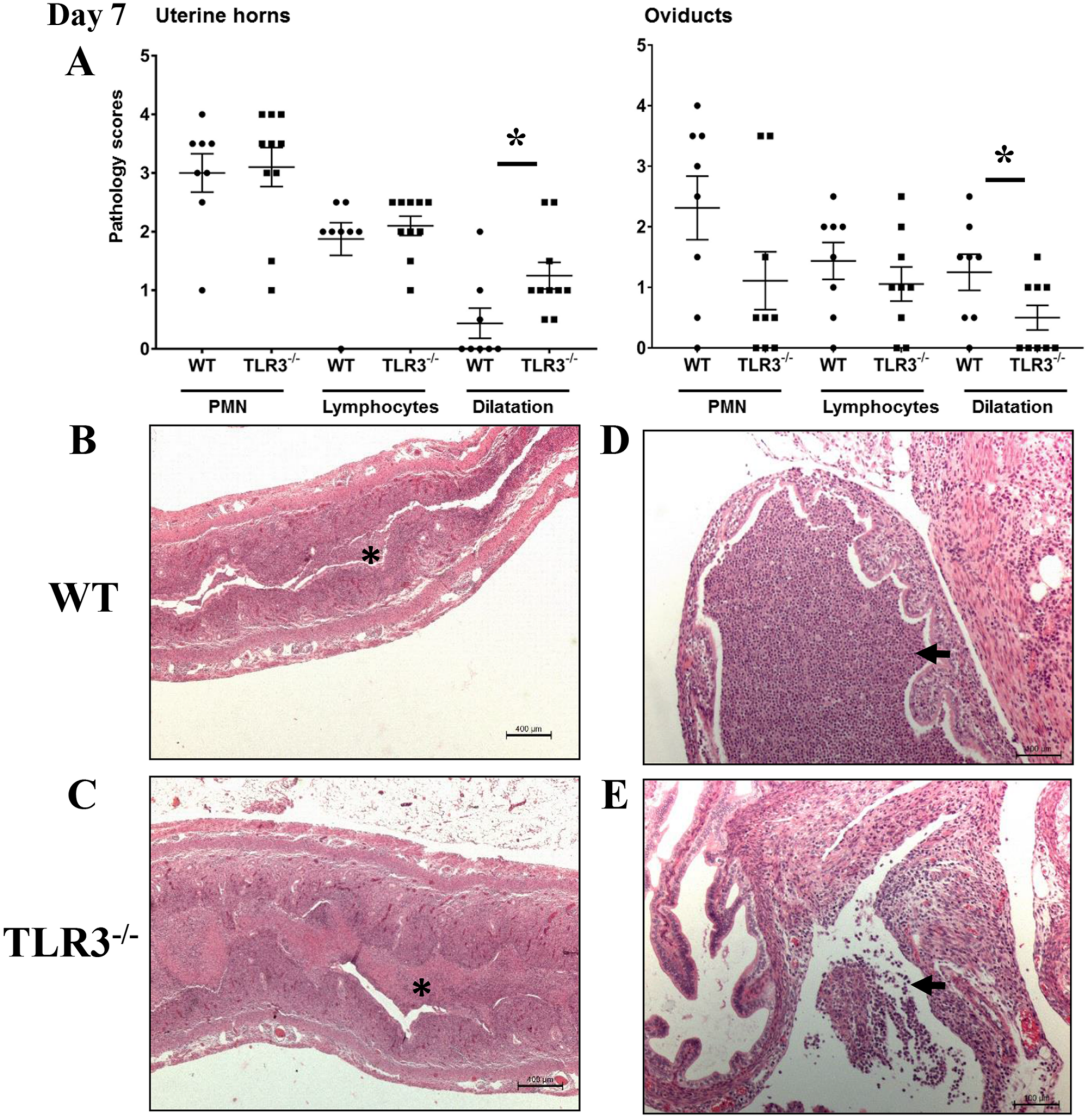
Acute inflammation in uterine horns and oviducts of wild-type (WT) and TLR3 deficient (TLR3^-/-^) mice on day 7 of *Chlamydia* infection. **A)** The extent of histopathological changes of both uterine horns and oviducts were scored using a four-tiered semi-quantitative scoring system in a double-blind fashion. These results were representative of 8–10 mice per group from three independent experiments. Differences between groups for each parameter were determined by two-tailed Mann-Whitney test (* = *p* < *0.05*). Only significant results are displayed in graphs. **B-C)** Histopathological evaluation of uterine horns from WT and TLR3^-/-^ mice shows that PMNs were commonly infiltrating the lumina of the uterine horns (asterisk) in both mouse strains. Uterine horn dilatation was frequently noted in infected TLR3^-/-^ mice (scale bar 100μm, 25x magnification). **D-E)** Histopathological evaluation of oviducts from WT and TLR3^-/-^ mice on day 7 of infection revealed mild oviduct dilatation in WT mice, but minimal oviduct dilatation in TLR3^-/-^ mice (scale bar 400μm, 100x magnification). There were PMN clusters in the lumina of oviducts (arrow). No significant differences of neutrophilic or lymphocytic infiltration was noted between the genital tracts of WT and TLR3^-/-^ mice. *Representative images for WT and TLR3*^*-/-*^ *mice are shown*. *(• = WT mouse; ▪ = TLR3*^*-/-*^ *mouse)*.

Because TLR3^-/-^ mice exhibited increased genital tract shedding of *Chlamydia* throughout the first three weeks of infection, we next evaluated whether TLR3^-/-^ mice also developed more severe pathology in the genital tracts during *Chlamydia* infection. Groups of 5 wild-type and TLR3^-/-^ mice were intravaginally inoculated with *C*. *muridarum*, and histological lesions of their uterine horns and oviducts collected at days 7 and 21 of infection were scored as described in Materials and Methods. There were no significant differences in neutrophilic or lymphocytic infiltration in the uterine horns and oviducts between infected TLR3^-/-^ and wild-type mice (Fig 2A). The uterine horn from both strains were moderately inflamed with multifocal to coalescing dense intraluminal aggregates of degenerate and necrotic neutrophils mixed with karyorrhectic nuclear debris and fibrin (Fig 2B and 2C). The endometrial epithelium was mildly disrupted at multiple regions of suppurative inflammation. The underlying endometrial stroma along the uterine horns was predominantly infiltrated with small numbers of lymphocytes (Fig 2B and 2C). The uterine horns from TLR3^-/-^ mice were slightly distended, and this feature was more pronounced in TLR3^-/-^ mice (Fig 2A). Although neutrophilic inflammation in oviducts and periovarian spaces of wild-type mice was not significantly different to the same areas in TLR3^-/-^ mice, the extent and distribution of the neutrophilic inflammation in these regions were slightly enhanced in wild-type mice (Fig 2A). The oviducts, periovarian spaces, and ovarian bursa from wild-type mice were predominantly infiltrated with multifocal aggregates of neutrophils and small numbers of lymphocytes in a background karyorrhectic nuclear debris and eosinophilic proteinaceous fluid (Fig 2D). Conversely, the inflammatory cell infiltrates in oviducts, periovarian spaces, and ovarian bursa from TLR3^-/-^ were composed by multifocal small aggregates of neutrophils, individual lymphocytes, and small amounts of eosinophilic proteinaceous fluid (Fig 2E). The oviduct epithelium from both group of mice were minimally eroded at sites of exudative inflammation. Oviductal dilation was mild and most commonly noted in wild-type mice (Fig 2A).

**Fig 3:**
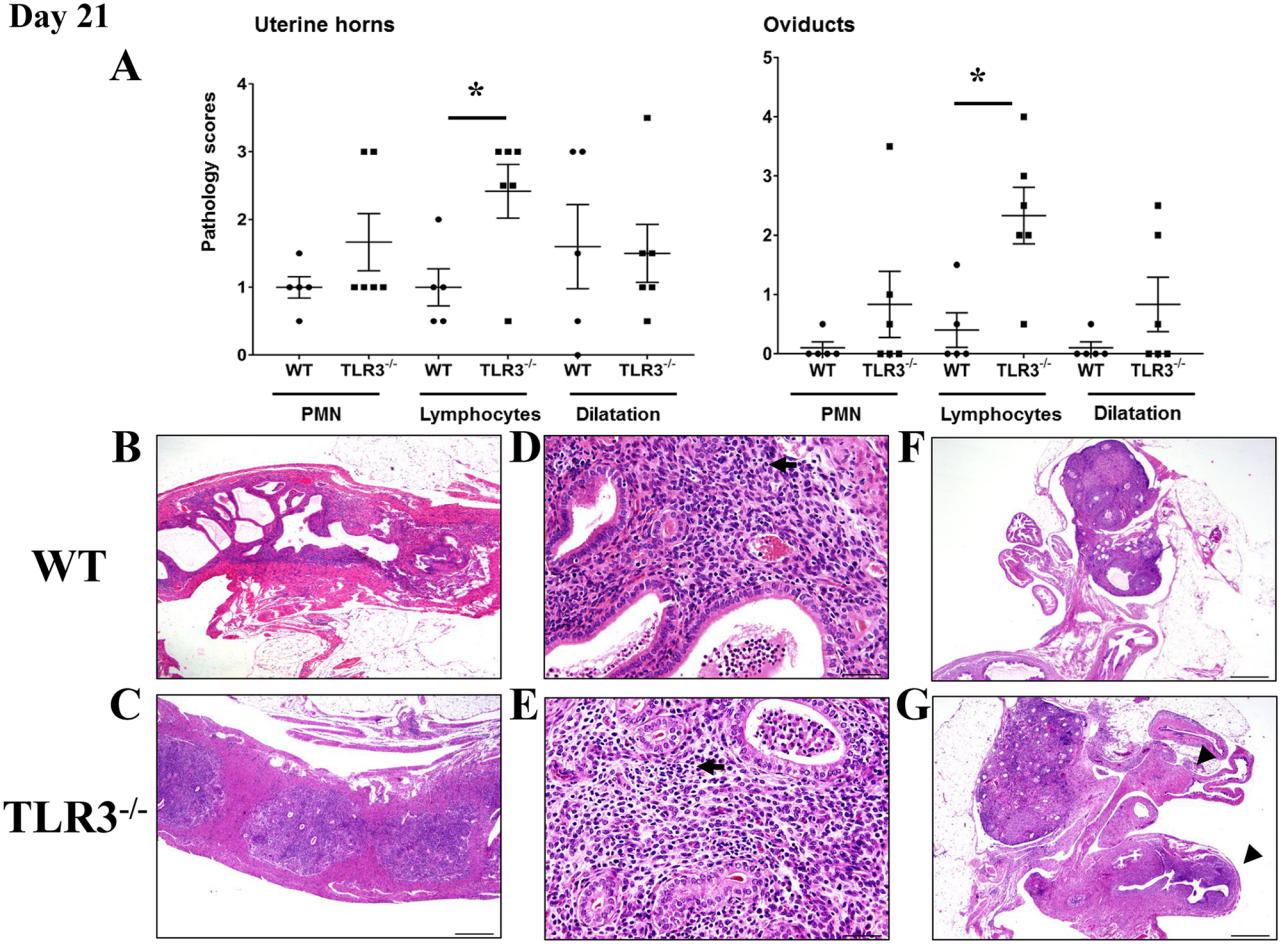
Lymphocytic endometritis and salpingitis is enhanced in the absence of TLR3 in mice during middle stage of *C*. *muridarum* infection. **A)** The extent of histopathological changes of uterine horns and oviducts were scored using a four-tiered semi-quantitative scoring system as described in Materials and Methods. These results were representative of 5–6 mice per group from two independent experiments. Differences between groups for each parameter were determined by two-tailed Mann-Whitney test (* = *p* < *0.05*). Only significant results are displayed in graphs. **B-E)** Histopathological evaluation of uterine horns from WT and TLR3^-/-^ mice showed that lymphocytes (arrows) were commonly infiltrating the endometrial stroma at day 21 of *C*. *muridarum* infection. Reproductive tracts of TLR3^-/-^ mice exhibited significantly higher lymphocytic inflammation than WT mice (A asterisk; B-E 20–400x magnification). F-G) Histopathological evaluation of oviducts from WT and TLR3^-/-^ mice showed that lymphocytic (arrowhead) inflammation was commonly found within the oviducts of TLR3^-/-^ mice (G 400x magnification). No significant differences in PMN infiltration was noted between the genital tracts of WT and TLR3^-/-^ mice. *Representative images for WT and TLR3*^-/-^ *mice are shown*. *(• = WT mouse; ▪ = TLR3*^*-/-*^ *mouse)*.

The inflammatory cell infiltrate in the genital tract of wild-type and TLR3^-/-^ mice at middle stages (day 21 of infection) of *C*. *muridarum* infection was predominantly composed of lymphocytes when compared with mock-inoculated controls. As shown in Fig 3, the degree of lymphocytic inflammation in the genital tract of TLR3^-/-^ mice was significantly different to the inflammation noted in the genital tract of wild-type mice. Scattered throughout the endometrial and oviductal lamina propria and ovarian bursa of TLR3^-/-^ mice were small to moderate numbers of lymphocytes with variable amounts of intraluminal proteinaceous fluid and clusters of degenerate neutrophils (Fig 3A and 3D–3E). In contrast, the lymphocytic inflammation in the endometrium and oviducts of wild-type mice was minimal (Fig 3A and 3A–3C). The uterine horns and oviducts from both mouse strains were from minimally to mildly expanded in size (Fig 3A). Together, these findings suggest that TLR3 signaling plays a role in the recruitment of lymphocytes to the endometrium and oviducts during middle stages of *C*. *muridarum* infection in mice.

### TLR3 modulates cytokine production in the genital tract of C57BL/6N during the acute phase of *Chlamydial* infection

**Fig 4:**
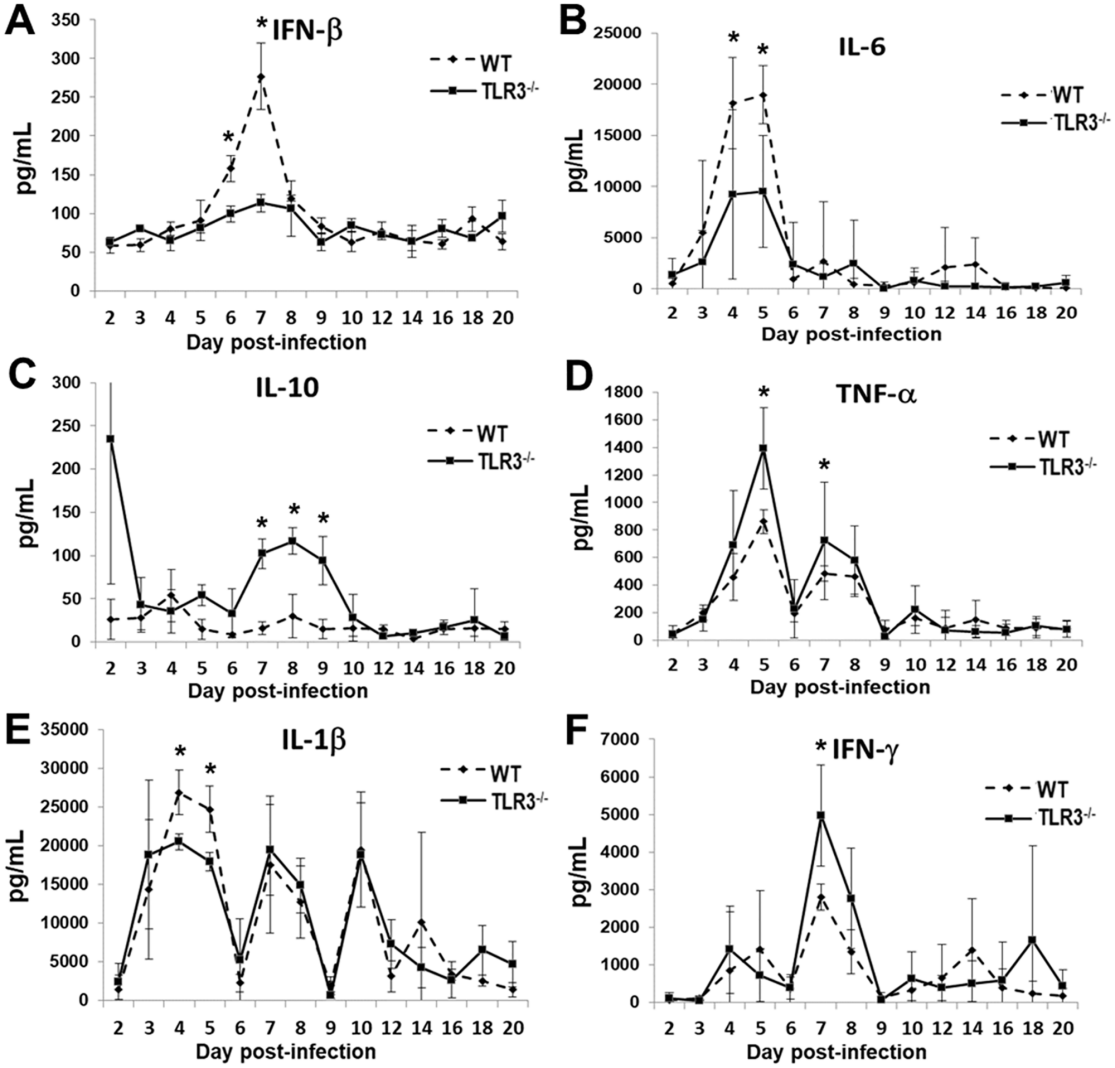
TLR3 modulates cytokine production in the murine genital tract during the acute phase of *Chlamydia* infection. Genital tract secretions were obtained during the first 20 days post-infection, and analyzed by multiplex cytokine assay for: (A) IFN-β, (B) IL-6, (C) IL-10, (D) TNF-α, (E) IL-1β, and (F) IFN-γ synthesis. Data are from a representative experiment where *n = 5* mice. * = *p* <*0.05*; ** = *p* <*0.005*.

Our previous findings have demonstrated that *C*. *muridarum* infection in OE cells induced the synthesis of IFN-β in a TLR3 dependent manner, and the synthesis of other inflammatory mediators, such as IL-6, CCL5, and CXCL10 were substantially diminished in OE cells isolated from TLR3 deficient mice derived from 129S1 genetic background [25, 26]. Moreover, the synthesis of IFN-β, IL-6, and CCL5 were diminished in the vaginal secretions of 129S1 TLR3^-/-^ mice infected with *C*. *muridarum* [25, 26]. These data suggest that oviductal epithelial cells play an important role in the synthesis of inflammatory mediators in the upper genital tract of mice during *C*. *muridarum* infection. To determine the significance that TLR3 has on the secretion of innate-immune cytokines and chemokines in the genital tract of C57BL/6N mice, groups of 5 wild-type and TLR3^-/-^ mice were intravaginally inoculated with 10^5^ IFU *C*. *muridarum* one week after treatment with Depo-Provera. As shown in Fig 4A and 4B, levels of IFN-β and IL-6 were significantly lower in TLR3^-/-^ mice, at day 7 and day 4–5 respectively (Fig 4A and 4B), corroborating with our earlier studies demonstrating that the 129S1-bred TLR3-deficient mice and TLR3-deficient OE cells were defective in the *Chlamydia*-induced synthesis of these cytokines (21, 23). IL-1β synthesized during early *Chlamydia* infection was also significantly diminished in TLR3-deficient mice between days 4 and 5 (Fig 4E), but was virtually identical to wild-type mice after day 5 of infection. In contrast, the TLR3^-/-^ mice secreted higher levels of IL-10, TNF-α and IFN-γ than wild-type mice (Fig 4C, 4D and 4F) at day 7 of infection, suggesting that TLR3 deficiency did not result in a global downregulation in synthesis of cytokines during early stages of chlamydial infection. Genital tract secretion levels of CXCL1, IL-2, and IL-4 were also measured; however, we found that the expression levels for these inflammatory mediators were virtually identical between the wild-type and TLR3^-/-^ mice (data not shown). Collectively, these *in vivo* data suggest an important role for TLR3 in modulating the synthesis of cytokines in the genital tract of mice during the early stages of *C*. *muridarum* infection.

### CD4^+^ T-cells recruitment is significantly altered in the genital tracts of TLR3^-/-^ mice during mid-*Chlamydial* infection

**Fig 5:**
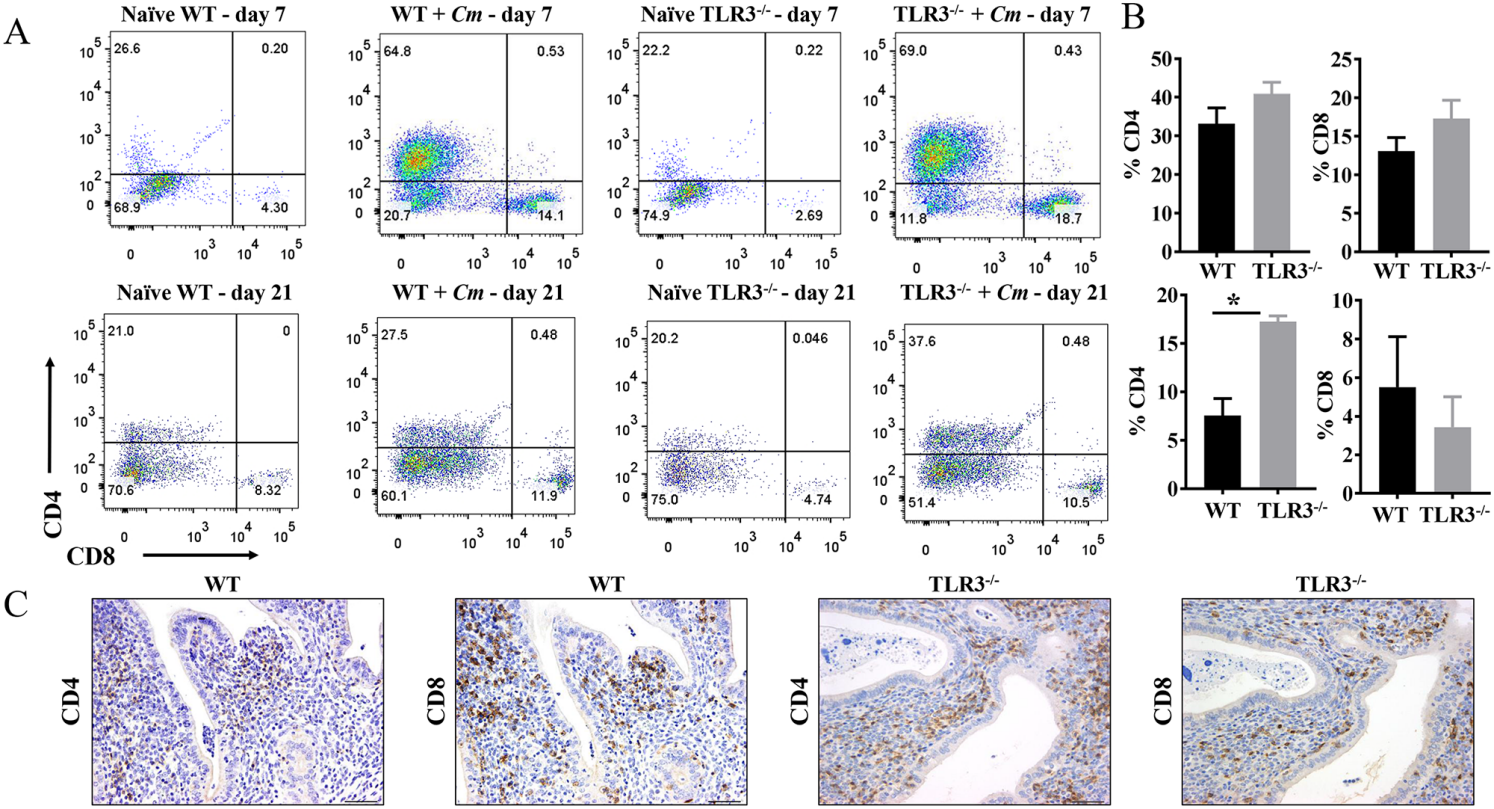
CD4^+^ and CD8^+^ T-cells recruitment into genital tracts of infected wild-type and TLR3^-/-^ mice. **A)** Groups of C57BL/6 and TLR3^-/-^ mice were infected intravaginally with 1×10^5^ *C*. *muridarum*. At day *7* and day 21 post-challenge, reproductive tracts from each group of mice were harvested, and lymphocytes were isolated as described in Materials and Methods. FACS plots showing percentage of CD4^+^ and CD8^+^ cells within the CD3^+^ T-cell population (gated on CD3^+^CD4^+^ or CD3^+^CD8^+^) in the genital tract of a mouse from each group. *FACS data showing representative results from each group of mice at days 7 and 21 of chlamydial infection*. **B)** The percentages of CD4 and CD8 T-cells from each group were summarized in the graphs. Data shown are representative results from two independent experiments with three to five mice per time point. Bar graphs show mean number ± SEM. * *indicate P values of* <*0.05*. **C)** Immunohistochemical staining of lymphocytes with antibodies to CD4 or CD8 in the genital tract of an infected mouse at day 21 of infection. Brown stain indicates CD4 or CD8 positive cells. *Original magnification ×200*.

In the experimental murine model for human intravaginal *Chlamydial* infections, CD4^+^ T-cells are required and sufficient to clear *C*. *muridarum* primary genital tract infections [5, 37–39]. Conversely, *Chlamydia*-specific CD8^+^ T-cell responses have been associated with scaring and immunopathology of upper genital tract infections in mice [40, 41]. We reported that TLR3-deficient OE cells and TLR3^-/-^ mice were defective in the *Chlamydia*-induced synthesis of several chemokines, such as CCL5, CXCL10, CXCL11, and CXCL16, that can act as chemo-attractants of T-cells *in vivo* [26, 42, 43]. Because the experiments above showed that the endometrial and oviductal stroma of TLR3^-/-^ C57BL/6 mice were significantly infiltrated with lymphocytes during middle stages of *C*. *muridarum* genital tract infections, we next evaluated the contribution of TLR3 on CD4^+^ and CD8^+^ T-cells in the genital tract of C57BL/6N mice. Groups of five wild-type and five TLR3^-/-^ mice were infected intravaginally with 10^5^ IFU *C*. *muridarum* before being sacrificed at either day 7 or day 21 of infection, and their lower genital tracts processed for flow cytometry as described in Materials and Methods. As shown in Fig 5, there were no significant differences in CD4^+^ and CD8^+^ T responses in the genital tracts between wild-type and TLR3^-/-^ mice during the early (acute) phase of *C*. *muridarum* infection (Fig 5A and 5B; Day 7). By day 7 post-infection, the CD4^+^ T-cell populations in the wild-type and TLR3-deficient mice were 64.8% and 69%, respectively, which represented an increased frequency of CD4^+^ T-cells in the genital tract of wild-type and TLR3^-/-^ mice was 38.2% and 46.8% over the mock controls. There were 14.1% and 18.7% CD8^+^ T-cells in the wild-type and TLR3-deficient mice, respectively, at day 7 post-infection, which indicated a 9.8% and 16.01% increase over mock controls. Compared to the total percentage of CD4^+^ T-cells present in the genital tract tissue at day 7, there was an approximate 3 to 4-fold reduction in the total frequency of CD8^+^ T-cells in the genital tracts of the wild-type and TLR3^-/-^ mice in the representative example. However, by day 21 post-infection, the frequency of CD4^+^ T-cells in the genital tract of wild-type and TLR3^-/-^ mice was significantly different (Fig 5B). As shown, the frequency of CD4^+^ T-cells in the genital tract of wild-type and TLR3^-/-^ mice was 6.5% and 17.4%, respectively. There were no significant differences on CD8^+^ T-cell recruitment in the genital tracts of wild-type and TLR3^-/-^ mice (3.58% vs 5.76%; representative example) during the middle stages of *C*. *muridarum* infection. Collectively, these results suggest that TLR3 may be involved in modulating the recruitment of CD4 T-cells required to clear *C*. *muridarum* from the genital tracts of C57BL/6N mice during middle stages of infection.

To illustrate the distribution of CD4 and CD8 T-cells in the genital tract of wild-type and TLR3^-/-^ mice at day 21 of *C*. *muridarum* infection, representative cross-sections of the genital tracts of these mice were stained by immunohistochemistry using anti-CD4 and anti-CD8 antibodies. CD4^+^ and CD8^+^ T-cell were frequently detected along the endometrial stroma and myometrium of uterine horns (Fig 5C) and occasionally infiltrating mucosal folds and smooth muscle of oviducts of wild-type and TLR3^-/-^ mice.

### TLR3 deficiency results in more pronounced hydrosalpinx in the upper genital tracts of mice at day 56 post-intravaginal inoculation with *C*. *muridarum*

**Fig 6:**
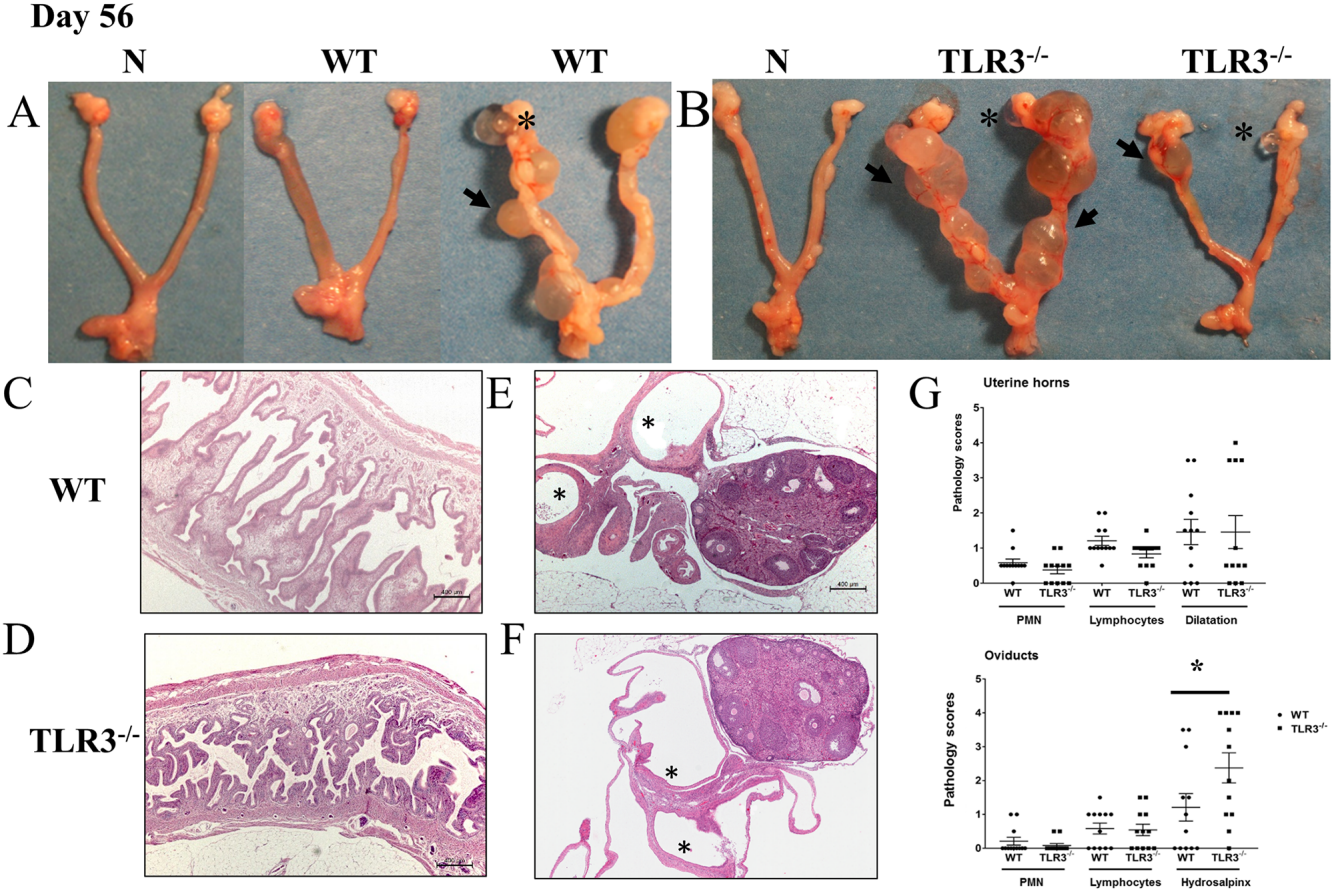
Hydrosalpinx is more commonly detected in TLR3^-/-^mice. **A-B)** Representative genital tracts removed from groups of wild-type mice (left) and TLR3^-/-^ mice (right) at day 56 of infection. Grossly, uterine horns were regionally or diffusely dilated in a subset of WT and TLR3^-/-^ mice. Oviducts and ovarian bursa were mildly to severely dilated (hydrosalpinx/ hydrobursitis) in subset of animals. **C-D)** Evaluation of uterine horns from WT and TLR3^-/-^ mice showed similar inflammatory changes characterized by small numbers of lymphocytic infiltration of the endometrial stroma with occasional neutrophils scattered throughout the uterine horn lumina. Uterine horns were partially filled with proteinaceous fluid with focal to multifocal endometrial cystic dilatation of glands that in some cases compressed and effaced the normal uterine wall architecture. **E-F)** Oviducts and ovarian bursa showed similarly mild inflammatory cell infiltrates in the oviducts, but significantly higher frequency and severity of hydrosalpinx in oviducts of TLR3^-/-^ mice (25x magnification, asterisks = hydrosalpinx). **G)** The extent of histopathological changes of both uterine horns and oviducts were scored as described in Materials and Methods. These results were representative of 12 mice per group from three independent experiments. Differences between groups for each parameter were determined by two-tailed Mann-Whitney test (* = *p* < *0.05*). Only significant results are displayed in graphs.

In the murine model of *C*. *muridarum* genital tract infections, the development of hydrosalpinx is used as one of the surrogate markers of chronic sequelae of infection and infertility [44]. Because TLR3^-/-^ mice exhibited more obvious lymphocytic endometritis and salpingitis during middle stage of chlamydial infection, we further evaluated whether TLR3^-/-^ mice also developed more severe hydrosalpinx in the upper genital tract. Groups of wild-type and TLR3^-/-^ mice were intravaginally inoculated with *C*. *muridarum*, and histological lesions in the lower and upper genital tract at day 56 of infection were scored as described in methods. There were no obvious differences in inflammatory cellular infiltrates in the uterine horns and oviducts of wild-type and TLR3^-/-^ mice at late stages of *C*. *muridarum* infection (Fig 6G). The endometrial and oviduct lamina propria (and associated smooth muscle) contain small numbers of lymphocytes and occasional neutrophils, with minimal erosion of the endometrial epithelial cells, an absence of intraluminal inflammatory cells, and variable degrees of unilateral and/or bilateral uterine horn dilation (Fig 6C, 6D and 6G). The periovarian space and oviducts from TLR3^-/-^ mice were mildly to moderately dilated (Fig 6A and 6B) with variable small amounts of intraluminal acellular, fibrillar mucinous fluid (hydrosalpinx). These oviductal changes were either bilateral or unilateral and more severe in oviducts of TLR3^-/-^ mice than oviducts of wild-type mice (Fig 6G). Dilated segments of the oviduct (e.g. ampulla, infundibulum and/or isthmus) were lined by an attenuated, intact surface epithelium. Additionally, the degree of periovarian dilation was commonly noted in oviducts with hydrosalpinx. Thus, these findings showed that TLR3 deficiency can lead to more pronounced chronic sequelae, such as hydrosalpinx, during late stages of *C*. *muridarum* genital tract infections in mice.

## Discussion

The pathogenesis of genital tract *Chlamydia* infections involves an interplay between chlamydial PAMPs and various host PRRs. This interaction during infection results in the release of various inflammatory mediators and chemokines that induce an influx of neutrophils into the genital tract, activate nearby macrophage and antigen presenting cells, and ultimately dictate the ensuing acquired immune response [3]. Toll-like receptors via MyD88 play an important role in host defense against chlamydial urogenital infections in mice by both mediating the recognition and early inflammatory response, and by steering the adaptive immunity toward a Th1 cell-mediated immune response [45, 46]. In addition, MyD88 independent pathways were also shown to be involved in modulating the inflammatory response during *C*. *muridarum* genital tract infections [25, 26, 47]. Our previous studies demonstrated that TLR3 signaling during *C*. *muridarum* infection plays a role in host defense and in the secretion of a variety of inflammatory mediators (including IFN-β) into the genital tracts of mice [25]. In this study, we expanded our investigations on the contribution of TLR3 in host defense and the immunopathology during *C*. *muridarum* infection in a congenic C57BL/6N mouse strain.

Our findings showed that TLR3 deficiency in congenic C57BL/6N and 129S1 mice [26] leads to a significantly increased shedding of *C*. *muridarum* during early and middle stage of genital tract infections. This enhanced efficiency of *Chlamydia* shedding was hypothesized to be attributed to the increased replication of *C*. *muridarum* in epithelial cells lining the genital tracts of TLR3^-/-^ mice. Our previous *in vitro* data support this hypothesis as *C*. *muridarum* growth in oviduct epithelial cells lacking TLR3 was more efficient when compared to wild-type OE cells, suggesting that stimulation of TLR3 by *C*. *muridarum* attenuates *Chlamydia* growth in these epithelial cells [26]. It is not yet clear whether the reduced replication of *C*. *muridarum* in wild-type OE cells are due to the direct interaction of *Chlamydia* factors with TLR3 in genital tract epithelial cells, or to a possible effect elicited by specific inflammatory mediators secreted during *Chlamydia* infection on the infected genital tract epithelial cells. Our *in vitro* data initially supported this later mechanism as IFN-β has an inhibitory effect on *Chlamydia* growth in oviduct epithelial cells [26]. However, our *in vivo* data showed that IFN-β likely modulates *Chlamydia* growth during early infection as IFN-β synthesis induced via TLR3 was simply elevated between day 6 and 8 of genital tract infections in wild-type mice. These findings suggest that additional host factors may modulate *C*. *muridarum* growth and survival in the genital tract of mice. For instance, it is possible that *Chlamydia* shedding is controlled by inducible nitric oxide synthase (iNOS) dependent mechanisms in the genital tract of wild-type mice. Investigations into the impact of iNOS dependent mechanisms on *Chlamydia* replication have shown that IFN-γ and T cell-epithelial cell contact results in upregulated iNOS genes and nitric oxide production, and these factors have been shown to be sufficient in controlling *C*. *muridarum* replication in genital tract epithelial cells [48, 49]. In support of this mechanism as a way to restrict chlamydial replication in these cells, other more recent studies have demonstrated *Chlamydia* replication in the genital tracts of mice is also modulated by iNOS independent mechanisms requiring T-cell degranulation [50, 51]. In addition, different lines of evidence have proposed that the interaction of *Chlamydia* with host cytoskeletal proteins plays a role in the release of infectious elementary bodies (EBs) from epithelial cells *in vitro* and *in vivo* [52–54]. Therefore, it will be of interest to determine whether TLR3^*-/-*^ genital tract epithelial cells have a different profile in cytoskeletal assembly and junctional proteins during *C*. *muridarum* infection, which may lead to an increased release of infectious EBs.

In view of the *Chlamydia* shedding findings above, we anticipated at the start of our investigation that TLR3 would protect against genital tract immunopathology. However, our study revealed the TLR3^-/-^ mice display comparable recruitment of inflammatory cells and/or disruption of the epithelium lining the reproductive tract (data not shown) with control mice during early genital tract infection. The enhanced dilation of the uterine horns in TLR3^-/-^ mice or the oviducts in wild-type mice during early stages of *C*. *muridarum* infection may be mediated by presence of neutrophilic-rich exudate and inflammatory mediators secreted as a result of *Chlamydia* replication at sites of genital tract infections [55, 56]. For instance, the recruitment of neutrophils into uterine horns and oviducts of infected mice is likely mediated by a variety of chemokines and/or proinflammatory cytokines, such as CXCL1, CXCL2, CXCL5, TNF-α, and IL-1β, which have shown to be produced during active *C*. *muridarum* infection in the genital tract of mice [27, 55–57]. We then showed that TLR3 deficiency resulted in the aberrant synthesis of several innate-immune factors including IFN-β, IL-1β, TNF-α, IFN-γ, IL-6, and IL-10 during the early stage of *C*. *muridarum* genital tract infection. TLR3 deficiency in mice led to increased synthesis of TNF-α, IFN-γ, and IL-10; however, IFN-β, IL-1β, and IL-6 were secreted into the genital tracts of wild-type mice at significantly higher levels than in TLR3^-/-^ mice during early infection. The impact that this differential expression of inflammatory mediators has on the overall outcomes of *Chlamydia* infection in TLR3^-/-^ mice is uncertain. However, it is possible that the increased secretion of TNF-α and IFN-γ in the genital tract of *C*. *muridarum* infected TLR3^-/-^ mice is in response to the relatively higher levels of *Chlamydia* replication during the first week of *C*. *muridarum* infection. In fact, our results on TNF-α synthesis were consistent with previous results showing that its synthesis is upregulated during the first week of *C*. *muridarum* infection in rodents [33, 58]. This may indicate that increased TNF-α levels enhances host defense in the C57BL6/N strain. Similarly, infectivity studies in IFN-γ-deficient mice from C57BL6 and 129S1 backgrounds [59, 60] demonstrated that IFN-γ played a major role in controlling *C*. *muridarum* genital tract infections in mice, and that CD4^+^ T-cells secreting gamma interferon (IFN-γ) were required for clearance of *C*. *muridarum in vivo* [5, 39, 61, 62].

The biological significance of the elevated synthesis of IL-10 in TLR3^-/-^ mice during early *C*. *muridarum* genital tract infections is unclear. IL-10 has been identified as a key player in the establishment and perpetuation of viral persistence, is known to dysregulate type-I interferon production, and promotes a suppressive environment that diminishes the antiviral response [63–65]. Also, there are numerous studies in *Chlamydia* addressing the hypothesis that changes in the regulation of this critical anti-inflammatory cytokine is associated with increased chronic disease complications and persistence in several different species of *Chlamydia* [66, 67]. Based on these findings regarding IL-10’s purported role in suppressing IFN-β, we can extrapolate a similar role for IL-10 in the suppression of IFN-β during *Chlamydia* infection in TLR3-deficient mice. Our data in Fig 4C shows that IL-10 was synthesized at significantly higher levels in the TLR3 deficient mice, but that it coincided at a time where there was peak IFN-β synthesis levels in the wild-type mice (Day 7, Fig 4A). Although it is unlikely that the increased IL-10 synthesis in TLR3-deficient mice impacts the *C*. *muridarum*-induced IFN-β synthesized early during infection (that we have shown to be mostly TLR3 dependent in OE cells), its synthesis more likely suppresses IFN-β secreted from pathways other than TLR3, which are mostly IFN-β amplification pathways that are active in the cell late during infection [68]. The proposed IL-10 mediated attenuation of IFN-β synthesis during TLR3 deficiency likely promotes the increased *C*. *muridarum* replication that we have observed in the TLR3-deficient mice and in our *in vitro* studies [26]. This hypothesis is supported in other studies where it was demonstrated that IL-10 synthesis was markedly upregulated in the lower genital tracts of *Cm* infected wild type mice, which resulted in a decreased Th1 response and increased chlamydial replication in the lower genital tract when compared to IL-10^-/-^ mice [67].

We also demonstrate that the TLR3-deficient mice show more signs of chronic disease complications such as lymphocytic endometritis and hydrosalpinx. Because IL-10 dysregulation is also possibly associated with outcomes of infection that are indicative of chronic sequalae of chlamydial disease [66], it is possible that the TLR3 signaling pathway either directly or indirectly regulates IL-10 synthesis as a way to limit genital tract pathology in the wild-type mice, and that putative regulation is absent in the TLR3-deficient mice. The proper balance between type-1 and type-2 IFN is critical in achieving the optimal immune response to *Chlamydia* infection, and dysregulation of that balance can have significant effect on outcomes of chlamydial disease [69]. Because our data show that IFN-β synthesis is diminished while IFN-γ synthesis is increased in the TLR3-deficient mice, we propose that this relative imbalance towards type-2 IFN in the genital tracts of infected mice may be one of the major reason IL-10 is more abundant compared to the wild-type controls. However, further study is needed to address the interplay between TLR3 signaling and IL-10 synthesis in order to better understand how dysregulation of IL-10 synthesis during *Chlamydia* infection of TLR3-deficient mice leads to more substantial genital tract sequalae.

Our *in vivo* findings have shown that *C*. *muridarum* infected TLR3^-/-^ mice generated in both the 129S1 and C57BL/6N backgrounds are defective in the synthesis of IFN-β on day 6 and 8 of infection, respectively. This data also support our previous findings of severely diminished IFN-β synthesis in TLR3^-/-^ OE cells [25, 26], which appeared to be a major contributor in the production of IFN-β *in vitro* [25]. Together, these findings suggest that *C*. *muridarum*, by a yet unidentified ligand, induces the expression of interferon regulatory factor 3 (IRF3) and IFN-β in a TRIF-dependent manner in oviduct epithelial cells during the acute phase of *C*. *muridarum* genital tract infection. Interestingly, other investigators have shown that IFN-β synthesis was downregulated during the first days of *C*. *muridarum* infections in IRF3^-/-^ mice when compared to wild-type mice, but the differential expression in IFN-β returned to normal levels after the first week of *C*. *muridarum* infection [70]. In addition, our previous *in vitro* studies showed that *Chlamydia* replication was more robust in the TLR3^-/-^ OE cells when compared to wild-type, and that pretreating the TLR3^-/-^ OE cells with IFN-β prior to infection significantly reduced chlamydial replication in these cells [26]. Our findings are suggestive that IFN-β has a detrimental role on *C*. *muridarum* replication during early genital tract of mice.

Similar to other investigators, IL-1β synthesis is markedly upregulated in the lower tract of C57BL6 mice during early *C*. *muridarum* infection, where the neutrophils and macrophages are thought to be the primary sources of this cytokine during infection [57]. The role and source of IL-6 synthesized during chlamydial genital tract immunopathology in mice is somewhat lesser defined and variable [71, 72]. IL-6 synthesis is upregulated in the genital tract of C57BL6 mice during the first week of *C muridarum* infection, and this process was initially shown to be independent of TLR4 and TLR3 [18, 26]. Furthermore, TLR2 has been proposed to induce the synthesis of IL-6 *in vitro*, as *Chlamydia* stimulates the production of IL-6 in murine oviduct epithelial cells and peritoneal macrophages in a TLR2 dependent manner [18, 73]. In a seemingly contrasting finding, our results here suggest that TLR3 signaling modulates the release of this pro-inflammatory cytokine during the first week of *Chlamydia* infection in mice, suggesting instead that its synthesis is TLR3 dependent. However, results of our earlier *in vitro* investigations into this phenomenon show that *Chlamydia*-induced TLR3-dependent IFN-β upregulates TLR2 gene expression, protein synthesis, and protein function in murine OE cells [26]. This interplay between TLRs 2 and 3 is representative of a possible regulation of TLR2-dependent immune responses by TLR3, and that this mechanism of regulation may be important to help control outcomes of *Chlamydia* infection in mice; particularly early- and mid-infection. It is likely that the TLR3 induced augmentation of TLR2 activity is absent early times during infection during TLR3 deficiency, and is what contributes to an aberrant immune response to *C*. *muridarum* in the TLR3 deficient mice.

Our findings showed that the endometrium and oviducts of TLR3^-/-^ mice exhibited significantly higher lymphocytic inflammation when compared to wild-type mice at day 21 post-infection. Furthermore, CD4^+^ T-cells in part contributes to this differential recruitment of lymphocytes in the genital tract of TLR3^-/-^ mice. A possible interpretation for this enhanced recruitment of CD4^+^ T-cells could be attributed to the increased burden of *Chlamydia* in the genital tract of TLR3^-/-^ mice, as CD4^+^ T-cells are essential in controlling *Chlamydia* burden from the genital tract mice [5, 39]. In addition, the kinetics of CD4^+^ T-cells in the genital tract of TLR3^-/-^ mice during the early and middle stages of infection is similar to what is observed during *C*. *muridarum* infections in BALB/c and C57BL/6 mice showing that Th1 T-cells remain high after 3 weeks post-infection [10, 74, 75]. The mechanism how TLR3 signaling recruits CD4^+^ T-cells during *C*. *muridarum* genital tract infection is currently unknown. Our studies studying the kinetics of lymphocytes during *C*. *muridarum* infection did not detect differences in total lymphocyte counts from peripheral blood and numbers of CD4^+^ T-cell in the spleen between TLR3^-/-^ and wild-type mice at day 7 and 21 of *C*. *muridarum* infection (data not shown). Thus, it is possible that TLR3^-/-^ mice displays an increased chlamydial-antigen-specific T-cell response derived from regional lymph nodes at later time points of genital tract infections. This finding is consistent with data from IRF3^-/-^ mice showing an enhanced proliferation chlamydial-antigen-specific T-cells derived from the iliac lymph node at day 14 and 21 of *C*. *muridarum* infection [70]. Alternatively, it is possible that *C*. *muridarum* infection in the genital tract of TLR3^-/-^ mice induces a more robust local expansion of chlamydial-antigen-specific T-cells when compared to wild-type mice. For instance, this enhanced CD4^+^ T-cells in the genital tract of TLR3^-/-^ mice may represent local expansion of tissue resident T-cells during *C*. *muridarum* infection in the genital tract of mice [76]. Further experimentation is required to determine the source of this enhanced proliferation of CD4^+^ T-cells in TLR3^-/-^ mice, and the chemokine/cytokine profile associated with this phenotype.

Our data showed that the incidence of oviduct hydrosalpinx is increased in the genital tracts of TLR3^-/-^ mice. Both the host’s innate and adaptive immunity have been purported to play a significant role in the development of upper genital tract pathology [77, 78]. In addition, the development of hydrosalpinx vary among different mouse strains from different genetic backgrounds and is independent to the duration and levels of *Chlamydia* shedding [79]. Previous studies have demonstrated that C57BL/6 exhibits the shortest course of *Chlamydia* shedding and markedly reduces development of hydrosalpinx at day 42 of chlamydial infection [33]. Similar to our experiments, no inflammatory infiltrates or fibrosis in the oviducts were detected in any of the groups of mice infected with *C*. *muridarum* at day 56 post-challenge. The exact mechanism on how TLR3 contributes to hydrosalpinx in mice remains enigmatic, but this lesion may have developed as a result of the chronic salpingitis from earlier time points of *C*. *muridarum* infection.

Our novel observations showing that TLR3 is stimulated during infection to exacerbate reproductive tract pathology in mice infected with *C*. *muridarum*, presents an interesting mechanism of bacterial-induced pathogenesis that may not be readily explained based upon our current understanding of TLR3 biology. Double-stranded RNA and possibly stathmin (a cellular protein that is a ubiquitously expressed regulator of microtubule formation [80]), are the only known ligands for TLR3. Our findings that TLR3 has such a prominent role in the pathogenesis of a bacterial pathogen not known to be associated with either ligand is both significant and enigmatic. One possible scenario that can provide some insight to this proposed TLR3-dependent mechanism of *Chlamydia* induced immunopathogenesis in mice intimates the possibility of a novel TLR3 PAMP (either bacterial or cellular) associated with *Chlamydia* infection. In this scenario, we propose that there is possibly a structural component of *Chlamydia* that is not likely a double-stranded RNA, since there is no known double-stranded RNA species associated with the chlamydial structure. We would hypothesize that this novel TLR3 PAMP associated *Chlamydia* could possibly be a chlamydial protein that has a similar α-helical structure of stathmin, and can potentially be identified via fractionation methodology [81].

Another possibility is that *Chlamydia* infection of reproductive tract epithelium somehow induces a cellular response resulting in the syntheses of cellular double-stranded RNAs, which could then bind-to and stimulate TLR3-dependent immune responses. The involvement of micro-RNAs (miRNAs) that are induced in response to bacterial infection has been investigated in gastric cancers during chronic *Helicobacter pylori* infection [82], and bacterial infections of the *Arabidopsis* plants [83]. In this scenario, we hypothesize the possible contributions of novel miRNAs that are endogenous to the epithelial tissue lining the reproductive tract, that are potentially induced during *Chlamydia* infection of these cells. In either scenario, the identification of the TLR3 PAMP that is presented during *Chlamydia* infection would represent a significant contribution to our understanding of TLR3 biology.

Overall, our results showed that TLR3 plays a role in host defense during the early and middle time points of infection in congenic C57BL/6N mice by modulating the production of proinflammatory cytokine at earlier time points, and by regulating the proliferation of CD4^+^ T-cells in the genital tract of mice at middle time points of infection. In addition, our data demonstrated the absence of TLR3 in the genital tract led to more severe symptoms of chronic inflammation and hydrosalpinx. Therefore, these findings suggest that TLR3 has a role in protection from uterine horn and oviduct pathology at middle and late time points of infection in mice. Greater understanding of the mechanism how TLR3 signaling modulates the development of upper genital tract pathology in mice may allow us to identify novel targets to reduce the risk of chronic sequelae caused by *Chlamydia* genital tract infections.
